# Supplementary material for: Individual interactions in a multi-country implementation-focused quality of care network for maternal, newborn and child health: A social network analysis
Source: PLOS Glob Public Health. 2023 Sep 21;3(9):e0001769. doi: 10.1371/journal.pgph.0001769 (PMC10513266; doi:10.1371/journal.pgph.0001769)
Supplement: S3 File — (DOCX) [file pgph.0001769.s003.docx]

**S3 File - Survey**

**Quality of Care Network - Social Network Analysis Survey [Country]**

**Introduction**

You have been invited to participate in this study because you have been involved with The Network for Improving Quality of Care for Maternal, Newborn and Child Health also known as the Quality of Care Network (QCN).

The objective of the study is to explore how the Quality of Care Network is enacted as a strategy for linking global and national actors. The aim of this social network analysis is to explore and describe the structure of the network. It will map the actors involved at health system different levels in order to understand the dynamics of interactions between actors globally as well as in countries (Bangladesh, Ethiopia, Malawi and Uganda).

In this social network analysis, we will try to map the networks for interaction, frequency of interaction, and information sharing among actors involved in the Quality of Care Network in Bangladesh, Ethiopia, Malawi, Uganda, and Global partners. This Social network analysis will allow us to visually present the interactions between partners. This should take about 20 minutes of your valuable time to complete. We are looking forward to see what we can learn. Thank you.

The QCN Evaluation group

**Consent**

Please note that your answers are confidential. Results that identify you by name will be kept within the research team. Anonymous codes will replace all names in the network maps. Note however that participation in this study is voluntary, and you may choose not to participate or withdraw your participation in this study at any time.

If you agree to participate in this study, you will be asked to answer questions about the people you interact with as well as some background information about you. To map out who interacts with whom, we will request you to give your name in completing this questionnaire.

**Your Consent**

I have read, and understood the introduction and the consent parts of this questionnaire.

- I understand the aim of the research project and what I will be asked to do
- I understand that I may stop my participation in this study at any time and that I can refuse to answer any questions.
- I also understand that if I take part in the Social Network Analysis I will be required to provide my name which will be replaced with a code.

| My Decision: I choose to: | |
| --- | --- |
| 1 | Participate in the study |
| 2 | Not to participate in the study |

**1. Your background**

| **a***.* What is your name? | | | | |
| --- | --- | --- | --- | --- |
|  | | | | |
| **b**. What is your sex? | Female |  | Male |  |
| *Note: Confidentiality and anonymity is guaranteed. No name will be mentioned in any report* | | | | |

**2.** What is your general professional background or training?

| General profession | *Mark with X* |
| --- | --- |
| - Doctor |  |
| - Nurse |  |
| - Midwife |  |
| - Information officer |  |
| - Pharmacist |  |
| - Laboratorist |  |
| - Manager |  |
| - Other (please specify) |  |

**3**. Within what level of the network are you primarily working?

| Primary level of engagement | *Mark with X* |
| --- | --- |
| - Facility level |  |
| - Subnational/regional level |  |
| - National level |  |
| - Global level |  |
| - Other (specify) |  |

4. What is your primary role in the network?

| Primary role in the Quality of Care Network | *Mark with X*  *all that apply* |
| --- | --- |
| - Frontline health worker |  |
| - Member of any related committee (national, subnational, facility or other quality related committee) |  |
| - Implementing partner |  |
| - Technical partner (providing technical support to those working in the Quality of Care Network) |  |
| - Other (specify) |  |

**5.** Please select the country you are representing in the Quality of Care Network

| - Bangladesh |
| --- |
| - Ethiopia |
| - Malawi |
| - Uganda |

6. Please select your current job/position title as relevant to your setting (by country)

| **What is your current job (cadre) in [country]?** ***(Tick/specify Here)*** | |
| --- | --- |
| Nurse |  |
| Midwife |  |
| Doctor |  |
| Health officer |  |
| Medical Laboratorist |  |
| Director |  |
| Deputy Director |  |
| Technical assistant/advisor |  |
| Quality advisor |  |
| Facility manager/head |  |
| Other (Please specify) |  |

**7-9**. The next set of questions include names of individuals working in the Quality of Care Network at different levels of the health system: local or subnational level, national level, and global level.

With your responses, we will map the network of interactions between partners in your country and with global/international partners.

**7. LOCAL AND SUBNATIONAL LEVEL**:

A list of people is presented to you. They work at the sub-national or local level in your country.

You are requested to please indicate if you interact (or have interacted) with the individual in matters relating to the Quality of Care Network and how often. Please also indicate the nature of your interactions specifically for Learning, Taking action, and Information sharing.

If you interact (or have interacted) with additional people, please add their names and respond to each question for each individual. You may add as many names as appropriate to respond to the questions.

*[****Note:*** *Please identify as many as possible and add names of any other person not listed - specify their location and role/function]*

| **Names** | **Profession or current job title** | **Affiliation/Facility** | **Primary level of engagement in network** | **Primary role in the network** | **Have you interacted with this individual on the Quality of Care Network?**  **(If yes, please check the box)** | **Please indicate how often you interact with this individual on matters related to the Quality of Care Network** | **Have you undertaken some *learning activities* related to Quality of Care Network with this individual?** | **Have you *taken forward actions* related to Quality of Care Network with this individual?** | **Have you *shared information* related to the Quality of Care Network with this individual?** |
| --- | --- | --- | --- | --- | --- | --- | --- | --- | --- |
| *(Fill in)* |  | *(Fill in)* | *Facility*  *Subnational*  *National*  *Global* | *- Frontline healthworker*  *- Member of any related committee*  *-Implementing partner*  *- Technical parter*  *Other (specify)* | *(If yes, mark with X)* | *1 = Never*  *2 = Annually*  *3 = Bi-annually*  *4 = Quarterly*  *5 = Monthly*  *6 = Weekly*  *7 = Daily* | (If yes, please check the box) | (If yes, please check the box) | (If yes, please check the box) |
| country specific details added | details provided | details provided |  |  |  |  |  |  |  |
| **[add others]** |  |  |  |  |  |  |  |  |  |

**8. NATIONAL LEVEL:** A list of people is presented to you. They work at the national level in your country.

You are requested to please indicate if you interact (or have interacted) with the individual in matters relating to the Quality of Care Network and how often. Please also indicate the nature of your interactions specifically for Learning, Taking action, and Information sharing.

If you interact (or have interacted) with additional people, please add their names and respond to each question for each individual. You may add as many names as appropriate to respond to the questions.

*[****Note:*** *Please identify as many as possible and add names of any other person not listed - specify their location and role/function]*

| **Names** | **Profession or current job title** | **Affiliation/Facility** | **Primary level of engagement in network** | **Primary role in the network** | **Please indicate how often you interact with this individual on matters related to the Quality of Care Network** | **Have you undertaken some *learning activities* related to Quality of Care Network with this individual?** | **Have you *taken forward actions* related to Quality of Care Network with this individual?** | **Have you *shared information* related to the Quality of Care Network with this individual?** |
| --- | --- | --- | --- | --- | --- | --- | --- | --- |
| *(Fill in)* | Profession or current job title | *(Fill in)* | *Facility*  *Subnational*  *National*  *Global* | *- Frontline healthworker*  *- Member of any related committee*  *-Implementing partner*  *- Technical partner*  *-Other (specify)* | *1 = Never*  *2 = Annually*  *3 = Bi-annually*  *4 = Quarterly*  *5 = Monthly*  *6 = Weekly*  *7 = Daily* | (If yes, please check the box) | (If yes, please check the box) | (If yes, please check the box) |
| country specific details added | details provided | details provided |  |  |  |  |  |  |
| **[add others]** |  |  |  |  |  |  |  |  |

**9. GLOBAL LEVEL:** A list of people is presented to you. They work at the global level or in other countries.

You are requested to please indicate if you interact (or have interacted) with the individual in matters relating to the Quality of Care Network and how often. Please also indicate the nature of your interactions specifically for Learning, Taking action, and Information sharing.

If you interact (or have interacted) with additional people, please add their names and respond to each question for each individual. You may add as many names as appropriate to respond to the questions.

*[****Note:*** *Please identify as many as possible and add names of any other person not listed - specify their location and role/function]*

| **Names** | **Profession or current job title** | **Affiliation/Facility** | **Primary level of engagement in network** | **Primary role in the network** | **Please indicate how often you interact with this individual on matters related to the Quality of Care Network** | **Have you undertaken some *learning activities* related to Quality of Care Network with this individual?** | **Have you *taken forward actions* related to Quality of Care Network with this individual?** | **Have you *shared information* related to the Quality of Care Network with this individual?** |
| --- | --- | --- | --- | --- | --- | --- | --- | --- |
| *(Fill in)* | Profession or current job title | *(Fill in)* | *Facility*  *Subnational*  *National*  *Global* | *- Frontline healthworker*  *- Member of any related committee*  *-Implementing partner*  *- Technical partner*  *-Other (specify)* | *1 = Never*  *2 = Annually*  *3 = Bi-annually*  *4 = Quarterly*  *5 = Monthly*  *6 = Weekly*  *7 = Daily* | (If yes, please check the box) | (If yes, please check the box) | (If yes, please check the box) |
| details provided | details provided | details provided |  |  |  |  |  |  |
|  |  |  |  |  |  |  |  |  |
| [add others] |  |  |  |  |  |  |  |  |

**10. Any additional information/thoughts you would like to share with the QCN evaluation group?**

# Supplementary file 2: Data validation questions

Box S2.1: Data validation questions

| - Q1. Looking at the graphic (including the names/roles of those individuals identified as central nodes), how closely does the SNA present the organogram? i.e. does the results of the SNA reflect what is happening in your setting regarding the identified central actors? - Q2. Do any of the data surprise you (e.g. some key actors identified or not as central nodes, or clusters of nodes)? - Q3. In consideration of the other qualitative data analysed, how would you interpret these results? Does it support your findings or not? |
| --- |
