# Supplementary material for: Individual interactions in a multi-country implementation-focused quality of care network for maternal, newborn and child health: A social network analysis
Source: PLOS Glob Public Health. 2023 Sep 21;3(9):e0001769. doi: 10.1371/journal.pgph.0001769 (PMC10513266; doi:10.1371/journal.pgph.0001769)
Supplement: S4 File — (DOCX) [file pgph.0001769.s004.docx]

**S4 File: Data validation questions**

- Q1. Looking at the graphic (including the names/roles of those individuals identified as central nodes), how closely does the SNA present the organogram? i.e. does the results of the SNA reflect what is happening in your setting regarding the identified central actors?
- Q2. Do any of the data surprise you (e.g. some key actors identified or not as central nodes, or clusters of nodes)?
- Q3. In consideration of the other qualitative data analysed, how would you interpret these results? Does it support your findings or not?
