# Supplementary material for: Individual interactions in a multi-country implementation-focused quality of care network for maternal, newborn and child health: A social network analysis
Source: PLOS Glob Public Health. 2023 Sep 21;3(9):e0001769. doi: 10.1371/journal.pgph.0001769 (PMC10513266; doi:10.1371/journal.pgph.0001769)
Supplement: S5 File — (DOCX) [file pgph.0001769.s005.docx]

**S5 File: Additional results**

Table S4.1: Cross tabulation of actor characteristics in network

|  | **Frontline health worker** | **Implementing partner** | **Member of any related committee** | **Technical partner** | **Other/Not known** | **Grand Total** |
| --- | --- | --- | --- | --- | --- | --- |
| **Bangladesh network** | | | | | | |
| Facility level | 13 | 12 | 18 | 0 | 1 | 44 |
| Subnational | 0 | 2 | 3 | 0 | 5 | 10 |
| National | 3 | 9 | 5 | 7 | 17 | 41 |
| Global | 0 | 1 | 2 | 7 | 0 | 10 |
| **Grand Total** | **16** | **24** | **28** | **14** | **23** | **105** |
|  | | | | | | |
| **Ethiopia network** | | | | | | |
| Facility level | 33 |  |  |  |  | 33 |
| Subnational | 1 |  | 9 |  | 2 | 12 |
| National |  | 6 | 5 | 3 |  | 14 |
| Not known |  |  |  |  | 5 | 5 |
| Global |  | 2 |  | 23 |  | 25 |
| **Grand Total** | **34** | **8** | **14** | **26** | **7** | **89** |
|  | | | | | | |
| **Malawi Network** | | | | | | |
| Facility level | 97 |  | 35 |  |  | 132 |
| Subnational level | 6 | 1 |  |  |  | 7 |
| National Level |  | 2 | 7 | 5 | 2 | 16 |
| Global |  |  | 1 |  |  | 1 |
| not known |  |  |  |  | 6 | 6 |
| **Grand Total** | **103** | **3** | **43** | **5** | **8** | **162** |
|  | | | | | | |
| **Uganda** | | | | | | |
| Facility level | 99 | 0 | 39 | 0 | 0 | 138 |
| Subnational | 0 | 0 | 30 | 0 | 0 | 30 |
| National | 0 | 13 | 12 | 14 | 2 | 41 |
| Global level | 0 | 0 | 0 | 2 | 0 | 2 |
| **Grand Total** | **99** | **13** | **81** | **16** | **2** | **211** |
| **Global network** | | | | | | |
| Country level | **-** | 24 | 19 | 12 | **-** | 55 |
| Global level | **-** | 4 | 4 | 40 | **-** | 48 |
| **Grand Total** | **-** | **28** | **23** | **52** | **-** | **103** |

**Total nodes, potential interactions, reported interactions, and network density by network**

|  | Bangladesh | Ethiopia | Malawi | Uganda | Global |
| --- | --- | --- | --- | --- | --- |
| Nodes | 104 | 89 | 162 | 211 | 103 |
| Potential interactions/ Maximum number of ties | 10712 | 7832 | 26082 | 44310 | 10506 |
| Total interactions reported | 498 | 388 | 449 | 702 | 324 |
| Density | 4.6% | 5.0% | 1.7% | 1.6% | 3.1% |

Top 10 actors by network

Figure : Nature of interactions by country
