# Supplementary material for: Individual interactions in a multi-country implementation-focused quality of care network for maternal, newborn and child health: A social network analysis
Source: PLOS Glob Public Health. 2023 Sep 21;3(9):e0001769. doi: 10.1371/journal.pgph.0001769 (PMC10513266; doi:10.1371/journal.pgph.0001769)
Supplement: Attachment — Submitted filename: Response to reviewers.docx [file pgph.0001769.s007.docx]

Dear Editors at *PLoS Global Health*,

Thank you for considering this original research for publication in your journal. We appreciate the feedback from the reviewers and have made revisions to the manuscript incorporating their suggestions and feedback. We have also provided point-by-point responses to their comments, which is provided as a supplementary file.

We look forward to hearing from your regarding this revision.

Sincerely,
Mary Kinney on behalf of the authors

Journal Requirements:

1. Please include a complete copy of PLOS’ questionnaire on inclusivity in global research in your revised manuscript. Our policy for research in this area aims to improve transparency in the reporting of research performed outside of researchers’ own country or community. The policy applies to researchers who have travelled to a different country to conduct research, research with Indigenous populations or their lands, and research on cultural artefacts. The questionnaire can also be requested at the journal’s discretion for any other submissions, even if these conditions are not met.  Please find more information on the policy and a link to download a blank copy of the questionnaire here: <https://journals.plos.org/globalpublichealth/s/best-practices-in-research-reporting>. Please upload a completed version of your questionnaire as Supporting Information when you resubmit your manuscript.

Response: This questionnaire has been added to the Supporting Information.

2. We noticed that you used "unpublished" in the manuscript. We do not allow these references, as the PLOS data access policy requires that all data be either published with the manuscript or made available in a publicly accessible database. Please amend the supplementary material to include the referenced data or remove the references.

Response: We have updated these references.

3. We have noticed that you have uploaded Supporting Information files, but you have not included a list of legends. Please add a full list of legends for your Supporting Information files after the references list.

Response: We have added the legends for the Supporting Information files after the reference list.

4. We do not publish any copyright or trademark symbols that usually accompany proprietary names, eg  ©, ®, ™  (e.g. next to drug or reagent names). Please remove all instances of trademark/copyright symbols throughout the text, including ® on page 9.

Response: Thank you for informing us of this. We have removed the symbol.

Review Comments to the Author

Reviewer #1:

The paper addresses an important issue, the quality of care for mothers and children, through learning via networks. Social network analysis is a very interesting and promising method for this, since social networks reveal the potential and improvement areas for information sharing and learning, not only to the researchers, but also to the actors involved.

Overall, the data collected is extensive, probably extra challenging to do this across borders. The methods are very well adapted to local circumstances, using different data collection techniques, both on paper and digitally. This testifies to the persistence and perseverance with the researchers and certainly is worth a compliment.

However, the paper lacks preciseness and/or explanation in several respects, which makes it difficult for the reader to understand the relevance and implications of this study. I will go into that further per section of the paper, in the attached document.

Response: Thank you for your constructive feedback.

Title

1. Why use the term ‘organisational interactions’ when the social network reveals the individual’s interactions?

Response: We have removed ‘organisational’ from the title.

2. By definition information sharing takes place in case two actors interact. Whether this is information sharing is effective is not measured by SNA, let alone ‘learning’. Even though this was asked in the questionnaire I think that the evidence for learning is weak. I would suggest to focus this paper on interactions (which includes information sharing)

Response: Thank you for this comment.

**Individual interactions~~, learning and information sharing~~ in a multi-country implementation-focused quality of care network for maternal, newborn and child health: a social network analysis**

Introduction

3. The main elements of this paper are not described in much detail, and leave me with very basic questions like: what is this network about? What does it do? Who are ‘stakeholder groups? Is it one network or multiple networks? Why was it set up? Who are ‘groups of people’ (p. 69)? Why look at networks per country if QCN is described as (one) network (Line 244 suggests that there are multiple networks: ‘….For example…..as part of their network’.)?

Response: We appreciate this feedback and have added more details about the network in introduction. We have added a paragraph to the introduction including reference to the other papers in the Series, as proposed.

4. From what I understand this paper is one of a series. In the Discussion relevant findings from other papers in the same study context are mentioned. I think these should better be put in the Introduction, so it will become clear what this study adds to this.

Response: See above comment.

5. The ‘why’ of the network is important to understand, in particular the link with reducing mortality. Could you say more about (1) what are current mortality rates or stated ambitions by the stakeholders on this and (2) how is the network expected to contribute to lowering mortality rates

Response: We have added these details in the new paragraph that describes the network.

6. There is more to say from literature about how social networks – and how these are structured – contribute to network effectiveness, see for example the work of Provan (Networks within Networks) or Van der Ham (Integration and Differentiation in a Hospital’s Logistical System). In doing so it will be clearer what you are looking for in an effective social network (what could be effective and what is the function of SNA in this context?)

Response: Thank you for providing additional literature for us to consider to all us to clarify the scope of our work.

We have added these references including this sentence:

“For Provan and Julian, the effectiveness of a network (such as QCN) depends on the close coordination between mutually connected network subsets (cliques) where services are provided by differential categories of health professionals (van der Ham reference).”

7. There are more types of networks than dense and less dense, namely centralized, decentralized etc.

Response: We have added this sentence:

Other types of networks based on the various social interactions include: centralized vs decentralized networks, and integrated vs. segmented networks (Scott & Carrington reference). From this typology four other types of networks can be generated: (i) *“wheel/star*” networks (highly centralized and integrated); (ii) “*polycephalous*” networks (involving many clusters, with variable degrees of centralization); (iii) “*clique*” networks (totally decentralized and highly integrated); (iv) “*segmented, decentralized*” networks, comprising various components, made of horizontal cliques.

8. Box 1: I would put this in Methods, with an explanation of why you use these metrics (there are others as well, such as betweenness centrality that you don’t use, so please explain why)

Response: Thank you for the suggestion, Box 1 was moved to Methods section. We do agree that other SNA metrics exist as well, such as Betweenness centrality that measures how many times an actor sits on the shortest path between two other actors and put an emphasis on the actor’s control over information flow which was not the aim of this paper given the many domains explored.

9. A clear research goal and research questions are missing

Response: We have made this clearer and have added the research questions.

Methods

10. Line 113: there is a focus on density and indegree centrality, which is inconsistent with a focus on information sharing, collective learning and taking collective action (line 105-107). SNA only reveals how information is shared (see earlier remark on this)

Response: Thank you. We have clarified this issue in the introduction and methods. See response to point 2.

11. In the Study Setting the statement ‘conditions to be most favourable for the network in Bangladesh (line 122) should be in the Introduction I think. The way it is presented now is confusing with regard to this study, as these could be outcomes of this study.

Response: This has been moved to the introduction.

12. Study population and sampling: please explain more about ‘national and global levels’. What organisations are you talking about and included in this study? And what are national or global actors?

Response: We have added this information in the introduction when describing the networks as well made further revisions in the methods under study population.

13. Why ask/present gender if you don’t have a theory about it how this is relevant?

Response: Thank you for raising this point. A theoretical assessment of the gender relevance in this study was beyond our scope, as was trying to unpack and explain the different professional backgrounds of the respondents. That too would be interesting to further explore. We asked these general characteristic background questions to better understand the sample.

14. If (for example) 11 frontline workers are involved in the network from Bangladesh what do the 23 (34-11) doctors/nurses do in the network? Please explain more about the network

Response: Other papers in the collection go into more explanation of what the roles were of different health cadres in the network. It was beyond the scope of the study to understand what each individual did within the network. Our study was focused on their level and type of interactions.

Other papers in the collection go into more explanation of what the roles were of different health cadres in the network. It was beyond the scope of the study to understand what each individual did within the network. Our study was focused on their level and type of interactions.

See:

Shawar, Y., N. Djellouli, J. Shiffman, K. Akter, M. V. Kinney, K. Mwaba, G. Seruwagi, QCN Evaluation Group, M. English, T. Marchant and T. Colbourn (2023). "Factors Shaping Network Emergence: A Cross-Country Comparison of Quality of Care Networks in Bangladesh, Ethiopia, Malawi & Uganda. PLOS Global Public Health (under review).

or Medrxiv preprint 31st March 2023: doi: https://doi.org/10.1101/2023.03.29.23287925

https://www.medrxiv.org/content/10.1101/2023.03.29.23287925v1

Akter, K., Y. Shawar, A. Tesfa, C. Daniels-Howell, G. Seruwagi, A. Kyambulani, A. Dube, G. Gonfa, K. Mwaba, QCN Evaluation Group, M. V. Kinney, M. English, J. Shiffman, N. Djellouli and T. Colbourn (2023). "Influences on policy-formulation, decision-making, organisation and management for maternal, newborn and child health in Bangladesh, Ethiopia, Malawi and Uganda: the roles and legitimacy of a multi-country network " PLOS Global Public Health (under review).

or Medrxiv preprint 9th March 2023: doi: https://doi.org/10.1101/2023.03.06.23286634

https://www.medrxiv.org/content/10.1101/2023.03.06.23286634v1

Djellouli, N., Y. R. Shawar, K. Mwaba, K. Akter, G. Seruwagi, A. A. Tufa, G. Gonfa, K. Mwandira, QCN Evaluation Group, J. Shiffman, M. English and T. Colbourn (2023). "Effectiveness of a multi-country implementation-focused network on quality of care: delivery of interventions and processes for improved maternal, newborn and child health outcomes " PLOS Global Public Health (under review).

or Medrxiv preprint 5th March 2023 doi: https://doi.org/10.1101/2023.03.03.23286747https://www.medrxiv.org/content/10.1101/2023.03.03.23286747v1

15. Box 2: what are ‘learning activities’ and ‘forward actions’? How do respondents interpret this?

Response: The three domains – learning, action, and sharing information – were the QCN strategic objectives. As part of the QCN, the respondents would have had some introduction to these concepts and likely would have been actively participating in them. We intentionally left these terms undefined in the survey as this terminology should have been familiar to them as members of the network.

16. Line 148: the list of 20-30 names 🡪 how are names selected? Whose names are these?

Response: We have clarified that these names came from the results of the QCN evaluation study components that had already been underway when we did the SNA. These names were individuals who had already been identified as part of the network.

17. Line 171: what determines the direction of the arrow?

Response: We have explained this further: “The direction of the arrow (edge) goes from the ego (arrow tail) point to the alter (arrowhead), singling one respondent gave the name of an individual who they interact with in the network. Reciprocal relationships are displayed by a double-headed arrow.”

Results

18. Can you say something about the completeness of the network as revealed? With 566 actors identified, does this mean that 240 respondents involves approximately 43% of the total network?

Response: From Table 1: 240 were identified as sample respondents, but 302 responded to the survey which yield to 53% (302/566) network completeness with variations between cliques

We have added a sentence to read: “Across the five surveys, the 302 respondents identified 566 actors (or nodes) engaged with the QCN, ranging from 89 actors in Ethiopia to 211 actors in Uganda. This represented a 53% (302/566) network completeness, given that not all pairs of actors were connected.”

19. The concept of hubs is introduced in line 213, this is not a concept derived from SNA. Can you define this?

Response: This concept was described in the introduction following the network typology by Valdis and Holley. Hub refers to a central actor

20. What are ‘national actors’ (see also comment 12)

Response: As per our response on comment 12, we have clarified this in the methods.

21. Figures 2 are nice images of the networks, but they need more clarification to understand these:

There are orange nodes, but these are not in the legend. What is orange?

Table 3 and figures 2 are – at first glance – hard to match. For example for Bangladesh the top central actor is at subnational level, but from the figure 2a this is not visible. Is this actor B44? Please be more specific in the explanation. Also in figure 2b (Ethiopia) the top central actor appears to be orange (not being on national level). Please explain in the text for all five networks

What is the SNA metric used for ‘the most central actor’? In SNA this could be degree centrality or betweenness centrality

Response: The legend was updated, thank you. For Table 3 and Fig 2, thank you for this observation. We have updated with the correct legend to clarify and answer the question. Regarding your point on the SNA metric used, we have used the weighted degree centrality.

Discussion

22. The discussion contains a lot of new information, whereas it should reflect on results in relation to existing theory and/or previous research in this field. In line with comment 4 I would therefore suggest to shift a lot of the Discussion (results from previous research, lines 271-341?) to the Introduction.

Response: Thank you for this suggestion. We have made some changes to the introduction to include references to other studies in the collection; however, we feel that it is important to retain placing our results within their findings in the discussion rather than giving lengthy descriptions of their results in the introduction. We feel our approach avoids unnecessary repetition of both findings and other studies.

23. Also the discussed issues should reflect more extensively on existing theories (see comment 6) with regard to social networks, SNA and effective learning networks and maternal/child care.

Response: Thank you for the suggestion, We have added a sentence to reflect to the theory of network of care for maternal and child health and learning to read: “This study is in agreement with others that any network of care for maternal and child health can foster a multidisciplinary, multilevel teamwork, collaborative continuous learning, information sharing and problem solving”

24. In line 303 ‘observations from the field’ are mentioned, but in this study no observations are reported, neither in the Methods or the Results. If these are observations from previous studies, please describe these in the Introduction.

Response: We have clarified this statement.

Reviewer #2

This is an important contribution to the global health community given the use of multi-country collaboratives like the QCN. The social network analysis reveals some interesting gaps in how the network is working with significant room for improvement at global level and in the 4 countries.

Response: Thank you for reviewing the paper. We are glad you found it interesting and an important contribution to the literature.
