# Supplementary material for: Individual interactions in a multi-country implementation-focused quality of care network for maternal, newborn and child health: A social network analysis
Source: PLOS Glob Public Health. 2023 Sep 21;3(9):e0001769. doi: 10.1371/journal.pgph.0001769 (PMC10513266; doi:10.1371/journal.pgph.0001769)
Supplement: Attachment — Submitted filename: Second respond document PGPH-D-23-00370.docx [file pgph.0001769.s008.docx]

Response document

PLOS Staff Editor

Journal Requirements:

1. Please include a complete copy of PLOS’ questionnaire on inclusivity in global research in your revised manuscript. Our policy for research in this area aims to improve transparency in the reporting of research performed outside of researchers’ own country or community. The policy applies to researchers who have travelled to a different country to conduct research, research with Indigenous populations or their lands, and research on cultural artefacts. The questionnaire can also be requested at the journal’s discretion for any other submissions, even if these conditions are not met.  Please find more information on the policy and a link to download a blank copy of the questionnaire here: <https://journals.plos.org/globalpublichealth/s/best-practices-in-research-reporting>. Please upload a completed version of your questionnaire as Supporting Information when you resubmit your manuscript.

RESPONSE: WE HAVE COMPLETED THE QUESTIONNAIRE ON INCLUSIVITY.

Please review your reference list to ensure that it is complete and correct. If you have cited papers that have been retracted, please include the rationale for doing so in the manuscript text, or remove these references and replace them with relevant current references. Any changes to the reference list should be mentioned in the rebuttal letter that accompanies your revised manuscript. If you need to cite a retracted article, indicate the article’s retracted status in the References list and also include a citation and full reference for the retraction notice.

RESPONSE: WE HAVE GONE THROUGH ALL OF THE REFERENCES TO ENSURE NONE HAVE BEEN RETRACTED. FOR ARTICLES IN THIS SPECIAL SERIES THAT ARE STILL UNDER REVIEW, WE FOLLOWED THE SAME FORMAT AS THE PUBLISHED PAPER IN PLOS GLOBAL PUBLIC HEALTH HAS DONE.

<https://www.ncbi.nlm.nih.gov/pmc/articles/PMC10332621/>

Reviewer #2: A minor revision to Table 1. The number of respondents for Bangladesh is listed as 47 in row 3 whereas for the characteristics (gender, professional background, level of involvement and role), it adds up to 48. The authors should review and update for consistency.

RESPONSE: THANK YOU FOR NOTICING THIS ERROR. IT HAS BEEN CORRECTED TO 48.
