# Supplementary material for: Individual interactions in a multi-country implementation-focused quality of care network for maternal, newborn and child health: A social network analysis
Source: PLOS Glob Public Health. 2023 Sep 21;3(9):e0001769. doi: 10.1371/journal.pgph.0001769 (PMC10513266; doi:10.1371/journal.pgph.0001769)
Supplement: Attachment — Submitted filename: Rebuttal letter 10 August 2023.docx [file pgph.0001769.s009.docx]

**Rebuttal letter**

Comments from editor

RESPONSE IN CAPS

Thank you for correcting the number of Bangladeshi responses in Table 1 form 47 to 48. However, on page 7, line 2 (just above the table), the number of 47 is still given in the text.

Also, now the row totals does not add up correctly - I think it should be 303 rather than 302.

And the percentage for the corrected cell is now inaccurate (i.e., 48/55 is not 85.5% - it is 87.3%)

Please could you check all the numbers and percentages in the table and ensure they are accurate?

THANK YOU FOR YOUR CAREFUL CHECKING OF THE NUMBERS AND OUR APOLOGIES FOR MISSING THESE ERRORS. WE HAVE GONE THROUGH THE TABLE AND TEXT AND MADE ALL NECESSARY REVISIONS. THE REVISION WAS CHECKED FOR ACCURACY BY TWO AUTHORS LOOKING AT THE FINAL TABLES.
